# Beyond Screen Time and Emotion Regulation: Social Trust as a Structural Pathway to Perceived Well-Being—A Competing-Models Analysis Among Chinese Youth

**DOI:** 10.3390/bs16060847

**Published:** 2026-05-26

**Authors:** Jingyuan Zhang, Lanxin Su, Qiqing Xia, Sydney X. Hu

**Affiliations:** 1 Education Department, Kiang Wu Nursing College of Macau, Macau SAR 999078, China; mnhnu24029@stud.kwnc.edu.mo (J.Z.); sulx@hunnu.edu.cn (L.S.); mnhnu24021@stud.kwnc.edu.mo (Q.X.); 2 Health Science Center, Hunan Normal University, Changsha 410013, China

**Keywords:** social trust, perceived well-being, youth, well-being, screen time, social capital

## Abstract

Youth well-being interventions in digital contexts overwhelmingly target individual emotional mechanisms, yet whether these actually outperform social–structural pathways when directly compared has remained untested. Using nationally representative Chinese youth data (N = 1967; 91.4% of young adults; China Family Panel Studies 2022) and a competing-models structural equation framework with 5000-iteration bootstrap resampling, we simultaneously tested three rival pathways linking screen time to well-being. Results decisively favored the social–structural model: the social trust pathway yielded the strongest indirect association (β = −0.030, *p* < 0.001), despite the small size, nearly twice the magnitude of family-based mechanisms (β = −0.011, *p* < 0.001), while the direct emotional pathway was non-significant (β = −0.004, *p* > 0.05). Critically, negative emotions did not function as an independent parallel pathway; they emerged sequentially downstream of trust erosion. If emotional distress is downstream of trust erosion rather than a parallel input, interventions targeting emotion regulation address a symptom while the structural mechanism goes unaddressed. These findings suggest youth well-being interventions in digital contexts may benefit from rebalancing attention from individual behavioral modification toward social–structural conditions.

## 1. Introduction

Despite significant improvements in material living standards over the past two decades, the well-being levels of youth have shown a downward trend (Helliwell et al., 2024). This paradox of ‘happiness depression amid prosperity’ has sparked widespread attention in academia, yet existing theoretical responses appear overly simplistic. Previous explanations focus heavily on the negative impacts of digital technology (Twenge et al., 2018), arguing that excessive screen immersion triggers negative emotions, thereby reducing youth’s sense of well-being. This perspective presupposes a monotonic decline in well-being as screen time increases. Recent evidence suggests that the link between screen time and well-being may not be so straightforward (Chang, 2025). Beyond individual emotional manifestations, are there deeper, more fundamental structural factors associated with this phenomenon?

As a core component of social capital, social trust has long been regarded as a key predictor of well-being (Keyes, 2002; Wu & Kawachi, 2024). However, two interwoven trends are now gradually eroding this foundation. First, social trust has continued to decline in recent decades (Edelman Trust Institute, 2025; Rainie et al., 2019; Twenge et al., 2014), a trend further exacerbated by the widespread use of social media (Zhang & Zhang, 2024). Second, youth today spend approximately six hours daily on mobile devices (Manwell et al., 2022), fundamentally altering how social relationships are formed and sustained. The implications of these trends are profound: a 2025 World Health Organization report documents about 871,000 annual deaths attributable to social isolation (World Health Organization, 2025).

If social trust pathways account for more of the screen time–well-being relationship than emotional pathways do, then the field’s dominant intervention paradigm is targeting a secondary mechanism while ignoring the primary one. Public health increasingly recognizes the need to address root causes of health inequities rather than treating downstream symptoms (Marmot, 2005).

### 1.1. Competing Pathways Between Screen Time and Youth Well-Being

Contemporary well-being research has shifted from purely individualistic models toward social–ecological frameworks (Lomas et al., 2023, 2025; Pronk et al., 2025). The relationship between screen time and the well-being of youth still merits exploration (Bell et al., 2015; Santos et al., 2023). In the digital age, three distinct pathways could explain how screen time relates to well-being, each grounded in different theoretical traditions and pointing toward different interventions.

The emotional pathway reflects the dominant paradigm. Existing research indicates that screen exposure time is highly correlated with negative emotions (Khouja et al., 2019; Mougharbel et al., 2023) and is often accompanied by declining levels of well-being (Grisanzio et al., 2023). Recent high-powered studies have reinforced the emotional pathway’s credibility. Research from the United States documents that screen use among youth contributes to emotional distress, mediated by decreased physical activity and impaired sleep quality (Dai & Ouyang, 2026), and a 2025 meta-analysis confirms bidirectional amplification between screen exposure and socioemotional difficulties (Vasconcellos et al., 2025). This associative pattern constitutes the logical foundation of current policies: namely, if individual affect is a key dimension for understanding the relationship between the two, then interventions centered on emotion regulation should be effective. These findings establish that emotional mechanisms are real, replicable, and policy-relevant. The evidence base, however, is not without complexity: longitudinal associations between social media use and mental health difficulties have proven sensitive to platform type, measurement approach, and population characteristics (Cabezas-Klinger et al., 2025; Cheng et al., 2025; Odgers & Jensen, 2020; Vidal et al., 2020), further motivating a structural reanalysis. Furthermore, the generalizability of the relevant mechanisms beyond Western adolescent populations has not yet been sufficiently validated (Odgers & Jensen, 2020; Orben et al., 2022).

Yet a critical question remains unanswered by this literature: are emotional responses to screen time an independent mechanism or a downstream consequence of more upstream structural disruption? The dominant paradigm treats negative affect as a parallel pathway operating directly between screen exposure and well-being—an assumption rarely subjected to direct competitive testing. If emotional distress is, instead, sequentially downstream of trust erosion, then interventions targeting emotion regulation address a symptom rather than a cause. The present study does not contest the existence of emotional associations; it tests their relative position within a structural model that most prior work has not estimated.

The social trust pathway offers a structural alternative. Screen time leads to a phenomenon of ‘trust erosion’ by displacing social activities originally intended to build trust. This impact not only undermines interpersonal reciprocal relationships but also weakens public confidence in social institutions due to algorithmic curation and excessive exposure to institutional criticism. Trust erosion, in turn, reduces well-being by increasing the psychological costs of interaction and weakening support networks (Coleman, 1988; Putnam, 2000). Social trust provides a shared social resource that simultaneously supports cognitive evaluations of life and affective experiences in daily life (R. Li & Hu, 2026). Empirical studies confirm strong associations between trust and well-being (Bi et al., 2025; Guo et al., 2022; Lu et al., 2020; Xu et al., 2023; Yang et al., 2025), particularly in collectivist settings where trust operates through embedded networks (Q. Wang et al., 2023). Recent studies also link trust decline to negative emotional states (Kupferberg & Hasler, 2023; Liu et al., 2024), raising the possibility that emotions are downstream of trust rather than parallel to it.

The family pathway represents a micro-social mechanism. Screen time reduces engagement in household activities and caregiving (Madigan et al., 2019; Sbarra et al., 2019; Stiglic & Viner, 2019). Family engagement, in turn, provides protective mental health benefits (Feng & Tan, 2024; Vaivada et al., 2022). In collectivist cultures where family ties are particularly strong, this pathway could theoretically dominate.

### 1.2. Why China, and Why 2022

Social connection and trust face unprecedented erosion alongside rising digital technology saturation. The Commission on Social Connection documented 871,000 annual deaths attributable to social isolation (World Health Organization, 2025). China in 2022 offers a particularly informative test case. Due to the “dynamic zero-COVID” policy, youth in China experienced prolonged social restrictions, which intensified the dispersion of trust (Fang et al., 2023), while the country maintained the world’s highest rate of digital technology adoption (Montag et al., 2018). In this context, high digital penetration coupled with social restrictions allows for the examination of trust mechanisms.

Three limitations in existing research obscure social mechanisms. First, outcomes focus predominantly on psychopathology rather than well-being, despite well-being’s superior prediction of long-term health (Arslan, 2023; Holt-Lunstad, 2024). Second, sampling bias toward Western adolescents (ages 12–17) may amplify developmental sensitivities not generalizable to youth navigating trust-dependent life transitions. Third, theoretical frameworks privilege individual emotional mechanisms while treating social structure as mere context, yet emerging evidence from non-Western settings indicates social factors may supersede emotional ones (X. Li & Bian, 2024). This diagnostic tendency of reframing structural conditions as individual behavioral deficits has been identified as a broader pattern in health research (Gutin, 2024). Our findings further suggest that this pattern extends to the realm of digital health.

### 1.3. Study Objectives

**Figure 1:**
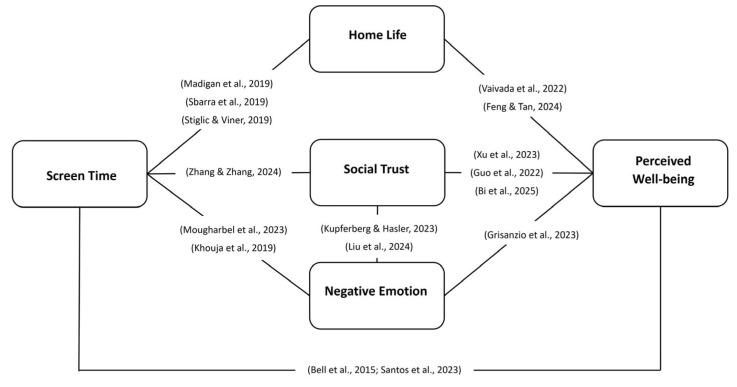
Conceptual framework. The family pathway links screen time to home life (Madigan et al., 2019; Sbarra et al., 2019; Stiglic & Viner, 2019) and home life to well-being (Vaivada et al., 2022; Feng & Tan, 2024). The social trust pathway links screen time to social trust (Zhang & Zhang, 2024) and social trust to well-being (Guo et al., 2022; Xu et al., 2023; Bi et al., 2025), with trust also relating to negative emotion (Kupferberg & Hasler, 2023; Liu et al., 2024). The emotional pathway links screen time to negative emotion (Khouja et al., 2019; Mougharbel et al., 2023) and negative emotion to well-being (Grisanzio et al., 2023). The direct pathway between screen time and well-being is also modeled (Bell et al., 2015; Santos et al., 2023).

To our knowledge, relatively few studies have explicitly examined which structural mechanism predominates in the screen time–well-being relationship, and empirical evidence remains limited regarding whether it outperforms emotional pathways when both are estimated within the same competing model. Using nationally representative Chinese youth data, this study addresses this gap through structural equation modelling with three directly competing pathways (Figure 1):

RQ1: Do social trust pathways show stronger indirect associations in the screen time–well-being relationship than direct emotional pathways?

RQ2: Do negative emotions operate primarily as downstream consequences of trust erosion (sequential mediation) rather than through direct parallel associations with screen time?

RQ3: Do social trust pathways exceed traditional family-based mechanisms in explanatory power?

## 2. Materials and Methods

### 2.1. Study Design and Analytical Framework

This study employed structural equation modeling to examine competing indirect association pathways between screen time and well-being among Chinese youth, using data from the 2022 China Family Panel Studies (CFPS). We adopted bootstrap resampling procedures with 5000 iterations to generate bias-corrected confidence intervals for all indirect association estimates (McRoberts et al., 2023).

### 2.2. Participants and Sampling

Our study utilized data from the 2022 China Family Panel Studies (CFPS). CFPS is a large-scale, multi-disciplinary longitudinal survey representative of the country. The 2022 survey covered 27,001 individuals of all ages and recorded a high response rate and data quality (Ethical review Approval Number: IRB00001052-14010). We selected people aged 16 to 35 as the youth sample and excluded cases with missing key variable data, total missing data reaching 20% or more, and abnormal key variable data. The final research sample included 1967 samples, with a balanced gender composition and urban-rural residential distribution.

#### 2.2.1. Inclusion and Exclusion Criteria

Inclusion criteria: Participants aged 16–35 years. This range aligns with the official definition of “youth” in China (spanning ages 14 to 35), as established by the CPC Central Committee and the State Council in 2017 (CPC Central Committee, 2017). The lower age limit was set at 16, primarily due to the survey design of CFPS, which administers personal self-report questionnaires for cognitive and psychosocial modules starting only at age 16.

Exclusion criteria: (1) Simultaneous missingness on more than three of the five key study variables (screen time, social trust, negative emotions, perceived well-being, and home life); (2) total missing data across all measured variables ≥20%; (3) screen time >20 h/day.

#### 2.2.2. Missing Value Handling

The details of variable missingness in this study can be found in Supplementary Table S1. Referring to the recommendations of Madley-Dowd et al. (2019) and Hughes et al. (2019), effective multiple imputation (MI) can reduce bias even when the proportion of missing data is high (Hughes et al., 2019; Madley-Dowd et al., 2019). The maximum missing rate in this study is 56.53% (<60%), so we used Iterative Imputation by Chained Equations (ICE) to handle missing data, implemented via the IterativeImputer module in Python 3.12.6. The algorithm utilizes Bayesian Ridge Regression as the base estimator to fill missing values through cyclic regression across variables. The maximum number of iterations was set to 10, and the random seed was fixed at 0 to ensure the stability and reproducibility of the results.

### 2.3. Measures

#### 2.3.1. Predictor Variable: Screen Time

Daily screen time (hours) was assessed by summing up two CFPS items: “ Internet usage time of mobile devices” and “ Internet connection time of computers “. This summary method conforms to the conventions for measuring screen time, as mobile devices and computers are the main online platforms for youth (M. Anderson & Jiang, 2018).

To control for demographic confounds (age, gender, educational attainment, and urban/rural residence) associated with both screen time and well-being, we employed the regression residual method (Cohen et al., 2003). First, observed screen time was regressed on demographic variables:

Screen time (predicted) = β_0_ + β_1_(age) + β_2_ (education attainment)  + β_3_(gender) + β_4_(residence) + ε

Secondly, the residual screen time was calculated by subtracting the predicted values from the observed values:

Screen time (residual) = Screen time (observed) − Screen time (predicted)

This residual variable represents the screen time variance orthogonal to demographic characteristics, thereby enabling a more accurate estimation of the unique association between screen time and the results (Greenland et al., 1999). The mediation analyses in this study all used residual screen time. This method is particularly suitable for cross-sectional exploratory studies, as complex covariate patterns may mask key associations (Judd et al., 2017).

#### 2.3.2. Outcome Variable: Perceived Well-Being

Perceived well-being was operationalized as a multidimensional construct encompassing subjective well-being (SWB) and psychological well-being (PWB), consistent with well-being theory distinguishing hedonic and eudaimonic dimensions (Keyes, 2002; Ryan & Deci, 2000).

Subjective well-being (SWB) was assessed using two validated scales: (1) the Self-Assessment Happiness Scale (Abdel-Khalek, 2006), which measures immediate emotional experience and global happiness on a single-item 1–5 scale (“In general, how happy do you feel?”), and (2) a Life Satisfaction Scale adapted from Andrews and Withey (1976), assessing cognitive evaluations of life quality across domains on a 1–5 scale (“How satisfied are you with your life as a whole?”) (Andrews & Withey, 1976).

Psychological well-being (PWB) was assessed using the Future Confidence Scale (Botella et al., 2018), capturing positive future expectations and sense of agency on a 1–5 scale (“How confident do you feel about your future?”). This measure reflects eudaimonic well-being’s emphasis on purpose and growth rather than momentary pleasure.

All items were standardized (z-scored) prior to aggregation to ensure equal weighting across scales with different response ranges. The composite well-being score demonstrated acceptable internal consistency (Cronbach’s α = 0.724), meeting conventional thresholds for social science research (α ≥ 0.70). Higher scores indicate greater perceived well-being.

#### 2.3.3. Intermediary Variables

Social Trust: According to Delhey and Newton, social trust can be categorized into particularized trust and generalized trust (Delhey & Newton, 2005). The former refers to trust in specific targets, such as kin or acquaintances (Zheng et al., 2023), while the latter represents a general expectation regarding the trustworthiness of ordinary others encountered in society (Robbins, 2023). Based on the results of exploratory factor analysis, this study selects trust in neighbors, local government officials, and doctors as measurement indicators (see Section 3.3). These indicators reflect attitudes toward ‘non-intimates’ in daily life and represent a dimension of social trust that extends from private relationships toward the community and the public sphere (Cronbach’s α = 0.681).

Negative Emotions: Negative emotional states were assessed using five items from the Center for Epidemiological Studies Depression Scale (Radloff, 1977). Prior research demonstrates that negative affect items from the CES-D show stronger reliability and validity than positive affect items when assessing emotional distress (Edwards et al., 2010). Based on exploratory factor analysis (see Section 3.3), we retained five items: “I felt down,” “I felt it was difficult to do anything,” “I felt lonely,” “I felt sad,” and “I felt that I could not go on.” Participants rated the frequency of each emotion during the past week on a 4-point scale: 1 (less than 1 day), 2 (1–2 days), 3 (3–4 days), or 4 (5–7 days). Items were summed (range: 5–20), with higher scores indicating greater negative emotional experience. Internal consistency was good (Cronbach’s α = 0.785).

Home Life: Time invested in family-oriented activities was measured by summing two CFPS duration items: daily hours spent on household chores and daily hours caring for family members. This composite variable reflects the time youth allocate to family-oriented activities in the home domain.

#### 2.3.4. Control Variable

Age (continuous variable, unit: years), gender (binary variable: 0 = male, 1 = female), educational attainment (continuous variable, years of education), and place of residence (binary variable: 0 = rural, 1 = urban) were included as demographic control variables. These variables are controlled by the regression residual method applied to screen time (see Section 2.3.1), thereby effectively eliminating the confounding associations between them and the predictor variables and outcome variables.

### 2.4. Statistical Analysis

Descriptive statistics, bivariate correlations, and exploratory factor analysis (EFA) were conducted using IBM SPSS 27.0.1. Structural equation modeling (SEM) was performed using IBM AMOS 26.0, following J. C. Anderson and Gerbing’s (1988) two-step approach: EFA to identify factor structures, followed by confirmatory factor analysis (CFA) to verify the measurement model (J. C. Anderson & Gerbing, 1988).

## 3. Results

### 3.1. Descriptive Statistics

Table 1 presents the demographic characteristics of the participants. The final sample comprised 1967 participants aged 16–35 years (M = 28.4, SD = 5.2). Our research sample is mainly composed of young adults aged 18 to 35, accounting for 91.4% of the total sample, which is in line with the age structure characteristics of the young population in China. This age distribution provides a sufficient sample basis for the subsequent sensitivity test.

Table 2 presents the descriptive statistics of the key variables. All variables presented acceptable normality (Brown, 2015), with skewness values ranging from −0.66 to 2.04 and kurtosis values ranging from 0.65 to 5.01.

Analysis of variance revealed significant age-related differences in screen patterns, as shown in Table 3 (F = 29.487, *p* < 0.001, df = 2). Usage peaked during emerging adulthood (18–25 years), with participants averaging approximately 7 h daily. Screen time then declined to around 6 h among adults aged 26–35. The average daily screen time was 6.00 h (standard deviation = 3.82).

### 3.2. Bivariate Correlations

Bivariate correlations among study variables are presented in Table 4. Social trust showed a strong positive correlation with perceived well-being (r = 0.36, *p* < 0.001), while negative emotions demonstrated a substantial negative correlation (r = −0.37, *p* < 0.001). Screen time showed non-significant correlations with both well-being (r = −0.002, *p* > 0.05). Moreover, social trust showed a negative correlation with negative emotions (r = −0.23, *p* < 0.001).

### 3.3. Exploratory Factor Analysis (EFA)

Data suitability for factor analysis was confirmed via Bartlett’s test of sphericity (χ^2^(78) = 5721.505, *p* < 0.001) and Kaiser–Meyer–Olkin measure of sampling adequacy (KMO = 0.814), exceeding the recommended threshold of 0.70 (Hair et al., 2013). Principal component analysis with varimax rotation revealed a cumulative variance explained of 59.79%, indicating satisfactory factor representativeness.

As for the Social Trust Factor Structure, principal component analysis of six trust items identified two interpretable factors (Table 5). The first factor, representing institutional and community trust, included trust in local government officials, trust in doctors, and trust in neighbors. These three items were retained for subsequent analyses, as they capture trust dimensions theoretically relevant to social capital (institutional and interpersonal trust). The second factor, representing particularized trust, included trust in strangers, but was excluded from analyses due to conceptual distinctness from generalized social trust.

As for the Negative Emotions Factor Structure, EFA of CES-D items identified a clear single-factor structure representing general negative affect (Table 6). The item “I don’t sleep well” was excluded due to low loading on the main factor, as sleep disturbance may reflect distinct physiological processes rather than affective states.

### 3.4. Confirmatory Factor Analysis (CFA)

#### 3.4.1. Measurement Model Fit

The measurement model demonstrated good fit, with χ^2^/df = 6.459, RMSEA = 0.053, GFI = 0.971, AGFI = 0.955, TLI = 0.925, CFI = 0.94, and IFI = 0.943. Although the χ^2^/df ratio exceeds the ideal range by 5, considering the sample size sensitivity of the χ^2^ test and given that other key fit indices all meet excellent standards, the overall model fit is acceptable (Alavi et al., 2020).

#### 3.4.2. Convergent and Discriminant Validity

The results of convergent validity are shown in Table 7. Results demonstrated adequate convergent validity with all latent variables achieving CR values exceeding the recommended 0.7 threshold.

Discriminant validity is presented in Table 8 with the square root of AVE for each latent variable exceeding its correlation coefficients with all other latent variables, confirming adequate discriminant validity among the constructs (Campbell & Fiske, 1959).

### 3.5. Structural Equation Model: Direct and Indirect Associations

#### 3.5.1. Direct Associations on Well-Being

**Figure 2:**
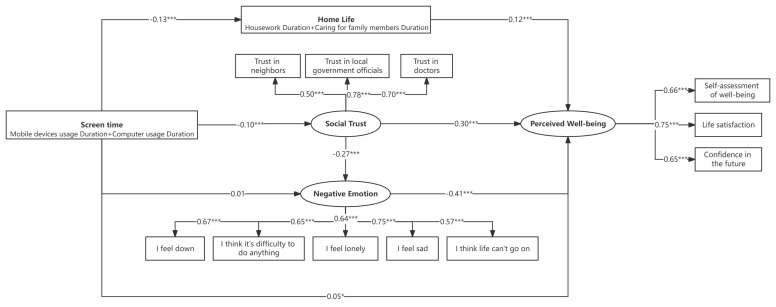
Structural equation model results. Note. * *p* < 0.05, and *** *p* < 0.001.

Figure 2 shows the standardized path coefficients of the structural model. Social trust (β = 0.30, *p* < 0.001, 95% CI [0.229, 0.380]) and family life engagement (β = 0.12, *p* < 0.001, 95% CI [0.075, 0.167]) were significantly positively correlated with well-being. Negative emotions, however, were significantly negatively correlated (β = −0.41, *p* < 0.001, 95% CI [−0.468, −0.341]). The intensity of the direct associations of social trust and negative emotions is comparable, indicating that the associations of these two factors with the well-being of youth are roughly equal, although the directions of action are opposite.

The direct association between screen time and well-being was small, positive, and statistically marginally significant (β = 0.05, *p* < 0.05, 95% CI [0.000, 0.093]). Its positive sign runs opposite to the negative indirect pathways, producing an inconsistent-mediation (suppression) pattern in which the direct and indirect associations partially offset one another. This offsetting is consistent with the near-zero bivariate correlation observed between screen time and well-being (r = −0.002, n.s.).

#### 3.5.2. Competing-Pathways Analysis: Trust-First vs. Emotion-First

Section 3.5.1 reported the direct associations of each predictor with well-being. In structural equation modeling, an indirect association is quantified as the product of the standardized path coefficients linking the predictor, the mediator(s), and the outcome (Hayes, 2013; Preacher & Hayes, 2008).

To test whether screen time is associated with well-being primarily through social trust (trust-first) or negative emotions (emotion-first), we simultaneously estimated four competing indirect pathways in a single structural model (Figure 2, Table 9).

Pathway 1: Trust-only pathway (the trust-first hypothesis)

The trust-mediated pathway was significant (β = −0.030, SE = 0.003, *p* < 0.001, 95% CI [−0.016, −0.005]): screen time was negatively associated with social trust, which in turn was positively associated with well-being.

Pathway 2: Emotion-only pathway (the emotion-first hypothesis)

In this single-mediator path, screen time is associated with well-being solely through negative emotions, with no involvement of trust (screen time → negative emotions → well-being). The indirect association along this pathway was non-significant (β = −0.004, *p* > 0.05, 95% CI [−0.008, 0.005]). This null result suggests that, at least in the present cross-sectional sample, the emotion-only pathway may be insufficient to account for the screen time–well-being link once trust mechanisms are included in the model.

Pathway 3: Sequential trust → emotion pathway

The sequential association (screen time → trust → emotions → well-being) was significant (β = −0.011, SE = 0.001, *p* < 0.001, 95% CI [−0.006, −0.002]), indicating that negative emotions showed sequential associations with lower trust levels rather than parallel associations. In this cross-sectional analysis, screen time was not directly associated with negative emotions; instead, the indirect pathway through social trust showed significant associations with well-being.

As shown in Table 9, the trust pathway (β = −0.030) was the most important indirect pathway and substantially exceeded the non-significant emotion-only pathway. Although home life exhibits a significant predictive role within the model, its strength of association is considerably lower than that of core variables such as social trust (β = −0.016). Combining the small positive direct association (β = 0.05) with these negative indirect associations yields a near-zero total association, consistent with the bivariate correlation reported in Section 3.2. This pattern indicates that indirect pathways offset the small positive direct association, supporting a trust-first mechanism.

### 3.6. Sensitivity Analysis

#### 3.6.1. Age Sensitivity Analysis

**Figure 3:**
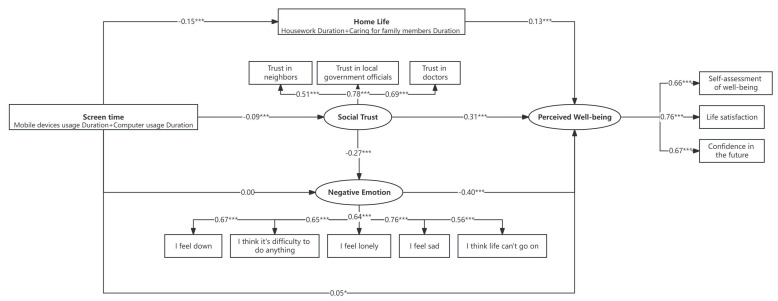
Age Sensitivity Analysis. Note. * *p* < 0.05, and *** *p* < 0.001.

Sensitivity analyses (Figure 3) excluding participants aged 16–17 years (n = 169) yielded patterns broadly consistent with those observed in the main analysis, lending support to the stability of our findings across developmental stages. The key structural paths showed similar magnitudes and directions: screen time was again positively associated with social trust (β = 0.11, *p* < 0.001), social trust again showed the strongest positive association with well-being (β = 0.30, *p* < 0.001), and negative emotions retained a strong negative association with well-being (β = −0.47, *p* < 0.001). Notably, the direct path from screen time to negative emotions remained non-significant (β = −0.07, *p* > 0.05), suggesting that the null emotional pathway is unlikely to be solely attributable to the inclusion of younger adolescents. The social trust indirect pathway also remained significant (β = −0.03, *p* < 0.001, 95% CI [−0.016, −0.004]), and a similar sequential mediation pattern (trust → emotion → well-being) was observed (β = −0.011, *p* < 0.001). Social trust showed a modest but significant negative association with negative emotions (β = −0.15, *p* < 0.01), and the direct association between screen time and well-being was small but significant (β = −0.07, *p* < 0.05). These consistencies indicate our findings are unlikely to be driven primarily by developmental heterogeneity and may reflect mechanisms that extend across young adulthood.

#### 3.6.2. Measurement Sensitivity Analysis

**Figure 4:**
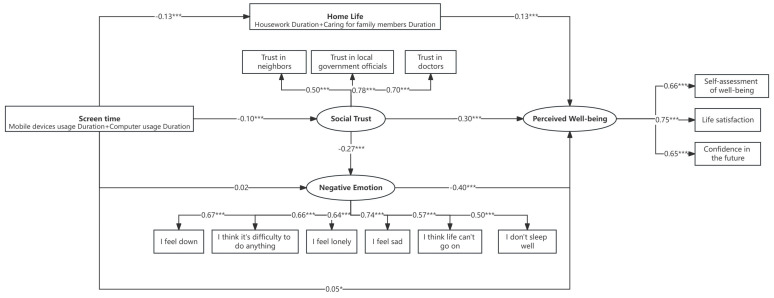
Measurement Sensitivity Analysis. Note. * *p* < 0.05, and *** *p* < 0.001.

To address potential concerns regarding the exclusion of the sleep disturbance item from the negative emotions scale, we re-estimated the structural model including all six original CES-D items (Figure 4). The core findings remained robust: the social trust indirect pathway maintained the same association size as the original model (β = −0.030, *p* < 0.001), the direct emotional pathway remained non-significant, and the sequential mediation pathway (β = −0.0112, *p* < 0.001) was comparable to the original model (β = −0.0108, *p* < 0.001).

## 4. Discussion

### 4.1. Principal Findings

This study identified social trust as the important mediating pathway linking screen time to well-being among Chinese youth, with three key findings challenging individual-behavior frameworks dominating digital health interventions. First, the social trust pathway (β = −0.030, *p* < 0.001) is the most important pathway, while emotional pathways were non-significant (β = −0.004, *p* > 0.5). Second, negative emotions operated sequentially through trust erosion (screen time → trust erosion → negative emotions → reduced well-being: β = −0.011, *p* < 0.001) rather than as parallel mechanisms, positioning emotional distress as a downstream consequence of social capital depletion. Third, the complete model explained 34% of well-being variance, providing certain evidence for understanding the transmission mechanism from screen time to well-being.

Prior work has called for upstream intervention but left unanswered which upstream factor matters most in digital contexts. The present findings provide that answer: when structural and emotional pathways compete directly, social trust dominates, while the emotional pathway’s independent effect becomes non-significant. These findings provide empirical support for recent theoretical work positioning social trust as a fundamental population health determinant (Wu & Kawachi, 2024), extending this framework into digital health contexts where trust formation faces unprecedented challenges from algorithmic curation, parasocial AI relationships, and platform-mediated interaction, lacking reciprocity essential to authentic social capital formation (Maples et al., 2024; Putnam, 2000).

### 4.2. Social Trust as Central to Well-Being in Digital Contexts

Social trust’s total association with well-being (β ≈ 0.41, combining direct and emotion-mediated pathways) exceeded all other predictors, including traditionally emphasized family factors. This finding supports the perspective that understanding the process by which social capital leads to instrumental or expressive returns, particularly health and well-being, is a critical step in leveraging social capital for health promotion purposes (Moore et al., 2013). This finding extends “Societal Trust-Mediated Well-being” pattern to digital contexts (R. Li et al., 2025; Liu et al., 2024) while challenging conventional perspectives that prioritize emotion regulation and hedonic experiences (Menefee et al., 2022).

Unlike the Western research that focuses on individual emotions, this study demonstrates the “social trust priority” model through the sequential mediating path of the Chinese sample: negative emotions often emerge after the weakening of social trust, rather than being directly caused by screen exposure itself. These findings are consistent with existing research: negative emotions do not arise directly, but are rather induced by the erosion of trust resulting from problematic social media use, with trust playing a critical mediating role (Lin et al., 2021). In the Chinese cultural context, a substantial body of research also confirms that the “weakening of trust” is a core antecedent of negative emotions (Z. Li & Yang, 2021; P. Wang & Zhang, 2025).

### 4.3. Screen Time as a Symptom

Our sequential mediation findings, combined with the non-significant direct emotional pathway, align with the compensatory internet use framework, which posits that when individuals experience a sense of frustration in their basic needs, particularly regarding relatedness, autonomy, and competence, they are driven to digital environments seeking compensatory coping strategies (Kardefelt-Winther, 2014). In fact, an increasing amount of evidence indicates that the social problems faced by youth and their low subjective well-being are closely related to the excessive use of problematic digital products (Twenge et al., 2021; Vasconcellos et al., 2025).

Screen time is likely to erode social trust as the most critical dimension of youth well-being in digital contexts through three interconnected mechanisms. First, opportunity cost displacement: time spent in digital environments reduces sustained neighborhood interactions and participation in local civic life. Although debate continues over whether social media directly displaces face-to-face interaction (Hall & Liu, 2022), the link between excessive digital media use and diminished offline social capital is well documented (National Academies of Sciences, Engineering, and Medicine, 2024).

Second, resocialization of interaction patterns: digital platforms train users toward interaction styles incompatible with authentic trust development. Algorithms optimize engagement over relationship depth; parasocial relationships with influencers replace reciprocal friendships (Hoffner & Bond, 2022; Schramm et al., 2024); quantified feedback (likes, follower counts) substitutes for nuanced recognition from sustained relationships (Bond, 2016). Youth spending critical developmental periods in environments prioritizing engagement metrics may develop expectations ill-suited to the mutual vulnerability required for trust-building.

Third, institutional trust erosion: sustained exposure to content depicting corruption or institutional failure undermines trust in social structures. Even a single exposure to critical social media content targeting public health institutions can damage trust (Lee, 2025). Adolescents relying heavily on social media for information often lack the ability to identify misinformation (Selnes, 2024). In rapidly modernizing societies where institutional legitimacy remains fragile, this mechanism may disproportionately shape youth’s developing trust orientations.

### 4.4. Implications for Intervention: Toward Trust-Centered Youth Well-Being in Digital Contexts

If screen time functions primarily as a compensatory symptom rather than a root cause, currently simple “digital restrictions” interventions (Radtke et al., 2022) may prove not merely ineffective but potentially counterproductive in the absence of concurrent trust-building support. Promoting well-being in digital contexts in an era of AI-mediated social interaction requires a paradigm shift from “digital detox” to “trust restoration. These findings extend “lifestyle drift” into the digital health domain (Williams & Fullagar, 2019), where intervention focus shifts toward individual emotional responses while leaving upstream social trust conditions unresolved. This diagnostic tendency of reframing structural conditions as individual behavioral deficits is a broader pattern in health research (Gutin, 2024) that also manifests in digital health. We propose a trust-centered youth well-being in digital contexts framework operating across three levels:

At the macro level, platform regulation could mandate algorithmic transparency and design features that promote genuine reciprocity rather than engagement maximization. Investing in offline civic infrastructure, such as community spaces, public programs, and participatory governance, will provide an alternative to digital-only social connections. Institutional accountability mechanisms could counteract the trust erosion amplified by digital misinformation ecosystems.

At the meso level, community-based programs could create repeated, trust-building social encounters through civic volunteering, community governance, or structured intergenerational activities (West et al., 2025). Workplace wellness programs could integrate social capital development alongside digital literacy, moving beyond screen time monitoring toward relational skill-building.

At the micro level, educational programs could teach youth to distinguish authentic reciprocal interaction from algorithmically optimized engagement, develop critical media literacy to counter institutional misinformation, and cultivate the self-disclosure and vulnerability needed for trust formation in environments that incentivize curated self-presentation.

### 4.5. Strengths and Limitations

Our research data were sourced from CFPS, with a large sample size and good national representativeness, providing high-quality and reliable data support for this study (Morgan & Winship, 2014). Although these findings advanced our understanding of the association between screen time and well-being, our research still had some limitations.

First, the cross-sectional design precludes causal inference; observed associations may reflect reverse causality (low well-being increasing screen time) or unmeasured confounding. Longitudinal designs tracking trust trajectories alongside digital behavior changes are needed. Second, our screen time measure (total duration) does not distinguish passive consumption from active engagement, without accounting for the type or purpose of digital engagement. Third, the findings are derived from Chinese youth during a specific historical period (2022, under zero-COVID restrictions); generalization to Western societies with higher baseline trust requires empirical validation. Fourth, this study has certain limitations at the measurement level. The items selected for social trust are somewhat skewed toward institutional trust, offering relatively limited representation of interpersonal trust. The home life composite measure captures the objective time that youth allocate to family-oriented activities, rather than their subjective experience of these activities (e.g., perceived burden or voluntary participation); accordingly, higher values do not necessarily indicate positive outcomes, as greater time investment in family life may potentially exert adverse effects on mental health. Fifth, although the direct and indirect pathways were statistically significant, the association sizes were small. Furthermore, because these effects operate in opposing directions, the bivariate association between screen time and well-being approaches zero. Readers should therefore interpret the practical significance of individual pathways with caution.

Future research should incorporate multidimensional trust assessments, longitudinal designs establishing temporal precedence, cross-cultural replications differentiating universal from context-specific mechanisms, and intervention studies testing whether trust restoration amplifies the protective effects of other resources as salutogenic theory predicts.

## 5. Conclusions

Our findings challenge the individual-behavior paradigm dominating global digital health policy, while simultaneously opening new directions for trust-centered well-being in digital contexts. Through competitive path analysis, we demonstrate that social trust erosion, rather than emotional dysregulation, is most significantly associated with the decline in well-being among Chinese youth. The sequential mediation pattern (screen time → trust erosion → negative emotions → well-being: β = −0.011, *p* < 0.001) suggests that emotional distress may reflect rather than drive well-being outcomes in digital contexts, repositioning negative affect as a downstream symptom of social capital depletion.

Importantly, these findings do not position digital technologies as uniformly detrimental. Platform features that promote genuine reciprocity, community co-creation, and transparent institutional communication hold potential as trust-building infrastructure rather than trust-eroding agents. The critical variable is not the time of usage, but the social architecture within which digital engagement occurs. Active, reciprocal digital engagement may reinforce the very trust networks that passive or algorithmically curated consumption tends to erode—a distinction with direct implications for both platform regulation and digital mental health intervention design. Prior calls for structural reorientation in digital health have lacked this specificity; this study provides it.

## Figures and Tables

**Table 1:** Demographic characteristics of participants.

Category	Group	Frequency	Percentage
Age	Late adolescent (16~17)	169	8.6%
	Emerging adult (18–25)	583	29.6%
	Established young adult (26–35)	1215	61.8%
Gender	Female	1020	51.9%
	Male	947	48.1%
Residence	Urban	1064	54.1%
	Rural	903	45.9%
Years of education	Illiteracy	25	1.3%
	Primary school	133	6.8%
	Junior high school	664	33.8%
	Senior high school	509	25.9%
	Higher education	636	32.2%

**Table 2:** Descriptive statistics for key variables.

Latent Variables	Mean	SD	Skewness	Kurtosis	Minimum	Maximum
Screen time(actual)	6.00	3.82	0.95	0.65	0.02	19.85
Home life	2.92	2.91	2.04	5.01	0.00	19.00
Social trust	19.20	5.00	−0.39	0.52	0.00	30.00
Negative emotion	7.64	2.45	1.06	1.58	5.00	20.00
Perceived well-being	15.52	2.86	−0.66	0.91	3.00	20.00

**Table 3:** Screen time usage by age group.

Age	N	Mean (h/Day)	Std. Error	Minimum	Maximum	95% Lower	95% Upper
Late adolescent (16~17)	169	4.99	3.36	0.03	13.43	4.48	5.50
Emerging adult (18–25)	583	6.96	3.94	0.02	19.85	6.64	7.28
Established young adult (26–35)	1215	6.00	3.72	0.02	19.79	5.47	5.88

**Table 4:** Bivariate correlations among latent variables.

	Screen Time (Actual)	Home Life	Social Trust	Negative Emotion	Perceived Well-Being
Screen time (actual)	1	-	-	-	-
Home life	−0.17 ***	1	-	-	-
Social trust	−0.07 **	−0.02	1	-	-
Negative emotion	0.04	0.18 ***	−0.23	1	-
Perceived well-being	−0.002	0.03	0.36 ***	−0.37 ***	1

Note. ** *p* < 0.01; *** *p* < 0.001.

**Table 5:** The component matrix after rotation of trust.

Item	Component	
	1	2
Trust in parents	0.287	0.000
Trust in neighbors	0.568	0.277
Trust in strangers	0.188	0.774
Trust in local government officials	0.883	0.215
Trust in doctors	0.794	0.026

**Table 6:** The component matrix after rotation of negative emotion.

Item	Component	
	1	2
I feel down	0.811	0.114
I think it’s difficult to do anything	0.712	0.244
I don’t sleep well	0.231	0.971
I feel lonely	0.701	0.174
I feel sad	0.762	0.174
I think life can’t go on	0.529	0.169

**Table 7:** Convergent Validity.

Latent Variable	Observed Variable	Std. Loading	UnStd. Loading	S.E.	C.R. (*t*-Value)	*p*	SMC	C.R	AVE
Perceived well-being	Self-assessment of happiness	0.66	1.00			***	0.44	0.73	0.48
	Confidence in the future	0.65	0.44	0.02	20.47	***	0.43		
	Life satisfaction	0.75	0.53	0.02	21.27	***	0.57		
Social trust	Trust in doctors	0.70	1.00			***	0.49	0.70	0.45
	Trust in local government officials	0.78	1.39	0.07	21.34	***	0.61		
	Trust in neighbor	0.50	0.73	0.04	17.07	***	0.25		
Negative emotion	I feel down	0.67	1.00			***	0.45	0.79	0.43
	I think it’s difficult to do anything	0.65	0.89	0.04	23.96	***	0.42		
	I feel lonely	0.64	0.87	0.04	22.91	***	0.41		
	I feel sad	0.75	0.97	0.04	25.72	***	0.57		
	I think life can’t go on	0.57	0.53	0.03	20.63	***	0.32		

Note. *** *p* < 0.001.

**Table 8:** Discriminant Validity.

	AVE	Screen Time	Social Trust	Negative Emotion	Home Life	Perceived Well-Being
Screen time	/	/	-	-	-	-
Social trust	0.45	−0.10	0.67	-	-	-
Negative emotion	0.43	0.04	−0.28	0.66	-	-
Home life	/	−0.13	0.01	−0.01	/	-
Perceived well-being	0.48	−0.01	0.41	−0.49	0.12	0.69

**Table 9:** Pathway Comparison.

Pathway	β	SE	*p*	95% CI	
				Lower	Upper
Direct associations with Well-Being					
Screen Time → Well-being	0.05	0.024	0.046	0.000	0.093
Social Trust → Well-being	0.30	0.038	***	0.229	0.380
Negative Emotions → Well-being	−0.41	0.032	***	−0.468	−0.341
Home Life → Well-being	0.12	0.023	***	0.075	0.167
Indirect associations					
Screen Time → Negative emotion → Well-being	−0.004	0.003	0.563	−0.008	0.005
Screen Time → Social Trust → Well-being	−0.03	0.003	***	−0.016	−0.005
Screen Time → Home Life → Well-being	−0.016	0.001	***	−0.008	−0.003
Screen Time → Social Trust → Negative Emotion → Well-being	−0.011	0.001	***	−0.006	−0.002

Note. *** *p* < 0.001.

## Data Availability

The public-use data employed in this study are available for download from the official China Family Panel Studies (CFPS) website (https://www.isss.pku.edu.cn/cfps/) upon user registration. Access to the restricted-use CFPS data referenced in this paper requires a formal application to the CFPS project team. Restricted data may only be accessed and analyzed within the designated CFPS data facility; the data are not publicly disclosed.

## Supplementary Materials

The following supporting information can be downloaded at: https://www.mdpi.com/article/10.3390/bs16060847/s1.

## Abbreviations

The following abbreviations are used in this manuscript:

CFPS	China Family Panel Studies
ST	Screen Time
WB	Well-being
NE	Negative Emotion
