# Supplementary material for: Beyond Screen Time and Emotion Regulation: Social Trust as a Structural Pathway to Perceived Well-Being—A Competing-Models Analysis Among Chinese Youth
Source: Behav Sci (Basel). 2026 May 26;16(6):847. doi: 10.3390/bs16060847 (PMC13296063; doi:10.3390/bs16060847)
Supplement: Supplementary file 1 [file behavsci-16-00847-s001.zip › behavsci-4294552-supplementary.pdf]

Supplementary Table S1. Variable missing situation

Latent variable	Observed variable	Missing rate
Perceived well-being	Self-assessment of happiness	0.05%
	Confidence in the future	0.00%
	Life satisfaction	0.00%
Screen time	Mobile devices usage duration	0.31%
	Computer usage duration	56.53%
Home life	Housework duration	42.09%
	Caring for family members duration	5.69%
